# Case-report: endovascular treatment of aortic pseudo-aneurysm caused by Fishbone

**DOI:** 10.1186/s13019-015-0304-z

**Published:** 2015-07-08

**Authors:** Wei Wang, Xuesong Liu, Mingjun Lu

**Affiliations:** Department of Cardiology, the First Affiliated Hospital of Guangzhou Medical University, No.151 Yanjiang West Road, Guangzhou, 510120 China

**Keywords:** Aortic pseudo-aneurysm, Interventional therapy, Stent grafts

## Abstract

Aortic pseudo-aneurysm (APA) is a rare disease in clinic. Because of its relative rarity, we are far from making any conclusion regarding the natural history and appropriate therapeutic strategy for this condition. This study is to investigate the treatment effect of interventional therapy in aortic pseudo-aneurysm. A woman of 68 years old diagnosed with APA caused by fishbone was treated with stent grafts. After treatment, the therapeutic effect was assessed by measuring the size of trauma. The patient recovered well after stent grafts treatment, as her trauma was minimal. However, some complications of intravascular interventional treatment were observed. Compared with conventional surgery, interventional therapy of intravascular stent grafts has its merits. Therefore, this strategy was worthy to apply in the treatment of aortic pseudo-aneurysm.

## Background

As one of the relatively fatal diseases, aortic pseudo-aneurysm always causes hemorrhage and thus threatens lives [1]. Main causes are trauma, infection, atherosclerosis or iatrogenicities [2, 3]. The conventional therapy is the aorta prosthetic vessel replacement, but featuring heavy trauma, higher incidence of complications and operative mortality. Introvascular graft exclusion has been evolving rapidly in recent years, and now gradually became the main therapy. We are describing here one case of treatment for the disease, providing insight into the natural course of this rare entity.

## Case Presentation

**Fig. 1:**
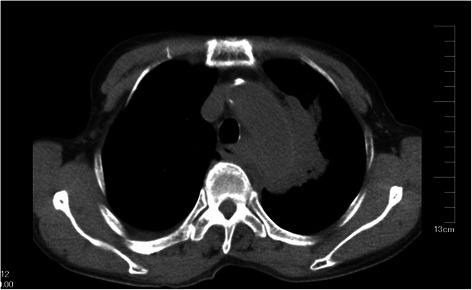
Radiological examination. One oval-like soft tissue density mass on aortic arch in upper right mediastinum

**Fig. 2:**
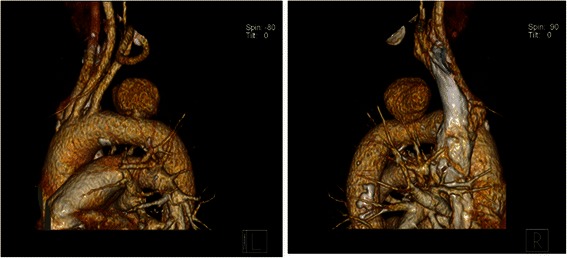
Three-dimensional computed tomography images for the shape of pseudoaneurysm on aortic arch, with a size of 2.9 × 4.3 × 2.6 cm. L: left side, R: right side

The patient was a woman of 68-year-old, and she was admitted in our hospital on 1^st^, August, 2011 due to pain in chest and back lasting for over 1 month. One month ago, the patient mistakenly swallowed down fishbone, and then felt pain and discomfort in throat, chest and back, and took vinegar later. On the second day, she visited Department of Otorhinolaryngology in local hospital. The fishbone was not seen after examination and pain in chest and back was not alleviated. The pain was dull and nonradioactive with unclear position, not aggravating significantly with change of body posture. Accompanied with little cough, expectoration, she had no hemoptysis, but palpitation, chest distress after exercises. The patient took Chinese herbs (unknown), but seeing little improvement and recurrent episodes of above-mentioned symptoms. On 28^th^, July, she was treated at Dongguan Hengli Hospital. A chest CT taken as part of a health examination showed that occupying besides upper left mediastinum; infection in middle lobe of right lung and left lingular lobe; and moderate volume of pleural effusion around both lungs. About 60 mL of yellow effusion was drained out with left pleural effusion drainage therapy. She had a history of hepatitis B for 6 years and two thyroidectomy operations for goiter in 2009 as diagnosed with thyroid adenoma. The enhancing 3-dimensional reconstruction of lung thin-layer CT scanning was shown in Figs. 1 & 2. One oval-like soft tissue density mass of 2.9 × 4.3 × 2.6 cm was detected on aortic arch in upper right mediastinum with unclear scanning border, which is suspected to be shaping of pseudoaneurysm on aortic arch. Moreover, organizing inflammation was observed around tumor and in left upper lobe; small amount of pleural effusion and nodular goiter were in two sides. The patient have experienced cardinal red hemoptysis (about 50 ml), and the symptom was alleviated after expectant treatment of hemostasis.

**Fig. 3:**
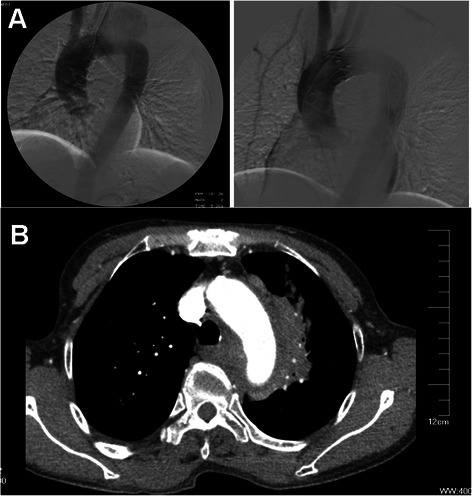
The determination of treatment effect by comparing. **a**. DSA angiography before intravascular graft exclusion for thoracic aortic pseudoaneurysm (Left) and angiography after operation (Right); **b**. Reexamination by Chest CT 6 days after operation

After multi-level diagnosis, it was likely that the aortic arch was injured by mistaken swallowed fishbone, and thus pseudoaneurysm was taking shape after fishbone penetrating esophagus and migrating into respiratory tract. We planed to block the channel between aorta and pseudoaneurysm with stent grafts so as to make a thrombotic tumor body that can be gradually organized and absorbed. On 5th, August, the patient received intravascular graft exclusion for thoracic aortic pseudoaneurysm: with general anesthesia, left radial artery was punctured with Seldinger's method. 6 F arterial sheath was implanted and Heparin (3000u) was injected into sheath; and then 5 F pigtail catheter was put into for aortography, showing sign of aortic arch pseudoaneurysm. The right inguinal region was cut layer by layer to expose the right femoral artery, and Amplatz Ultra Stiff Wire was put in; and then VALIANT THORACIC (30x200) was put in along Wire and then released to cover laceration. Aortography was carried out again, showing almost disappearance of pseudoaneurysm without leakage. Femoral artery and the incision were sutured layer by layer before covering wound with gauze (Fig. 3a). The operation was successful, and the patient had small amount of bloody sputum and low fever after operation. The therapy of controlling heart rate & blood pressure, anti-inflammation and hemostasis was followed up with gradual decrease of cough and bloody sputum. The body temperature was normal on the third day, and the pain in back and chest were gradually alleviated.

On 11th, August, Reexamination and Chest CT showed: stent was implanted in aortic arch and descending part, with no distortion; stent graft was well filled, and no stricture, dilation or filling defect were seen. Three branches of aorta were patent. Contrast media was not seen in original pseudoaneurysm (Fig. 3b). Esophagus barium meal: No foreign body was seen in hypopharynx and esophagus. The patient was improved greatly and discharged from hospital on 19th, August. Since the first follow-up, the patient felt no pain in the chest, but discomfort in chest after large amount of exercises, and felt better and no obvious discomfort after rest.

## Discussion

Causes of thoracic aortic pseudoaneurysm included the following five, trauma [4], postoperative complications [2, 5], atherosclerosis [6], infection [7, 8]. and Iatrogenicity, such as deep venous puncture [3, 8], others also include over dosage of anticoagulant drugs, etc. [9, 10, 11] Trauma, most are deceleration injury caused by car accidents, others also include high falling injury, heavy pound injury, sharp instrument injury, etc. Isthmus where the aortic arch and descending aorta meet is the most common happening part. Postoperative complications, including cardiac aortic operation, vascular reconstruction and angioplasty via skin puncture.

The conventional therapy for pseudoaneurysm is thoracotomy or prosthetic vessel replacement, featuring difficult operation, perioperative complications and high incidence of mortality. The operation requires extracorporeal circulation and whole body heparinization, associated organ injuries such as craniocerebral injury, and also possibly leads to craniocerebral hemorrhage and other visceral hemorrhage. If the descending aorta was blocked for a long time in the operation, renal insufficiency, paraplegia or myocardial infarction may be resulted in [12]. Attar [13] and other scholars reported that the morality and paraplegia of thoracotomy for traumatic pseudoaneurysm was 26 % and 11 % respectively.

Pseudoaneurysm is the most adaptable indication for stent-graft intravascular repair. Lacerations of pseudoaneurysm is relatively localized, and the upper and lower regions are normal arteries, which offer fine fixation for stent-graft. [14] Since the start of treatment of abdominal aortic aneurysm by Parodi in 1991 [15], and application of stent-graft for thoracic aortic aneurysm by Dake in 1994 [16], intravascular stent graft-graft for aneurysm has been becoming more of a concern to surgery and interventional physician due to its little trauma, slight hemorrhage, opener operation with more conventional operative indications, short operation time, short bed rest for patient, quick recovery, low intraoperative and postoperative morality, few complications as well as preventive treatment of complications. What is more, the postoperative life quality was greatly improved due to short hospitalization and less expenditure.

Since Dake first reported endovascular treatment for aortic dissecting aneurysm in 1994, Thoracic Endovascular Aortic Repair (TEVAR) has been widely used in clinics and been considered as a definitive solution in aortic dissecting aneurysm treatment. Although TEVAR would cause symptoms, including leakage, stent infection, retrograde aortic dissection and paraplegia, even death, the TEVAR still exhibited obvious advantages comparing to conventional thoracotomy. It is of safety, minimal invasion, quick recovery, few complications, and low mortality, which endowed the TEVAR to be a preferred solution in therapy of thoracic aortic aneurysm and aortic dissection.

Common complications included endoleakage, paraplegia, stent displacement, distal limbs embolization, and occlusion in stent. Endoleakage, as the worst complications for pseudoaneurysm, embolization of micro-endoleakage could be closed naturally after operation; but if the endoleakage were found in the operation of endovascular graft exclusion for pseudoaneurysm, it must be actively managed. Stent could be attached closely to vascular wall via balloon dilatation or reimplantation of a short stent to eliminate endoleakage. Precise location is needed for avoiding endoleakage, and then appropriate stent should be selected. For laceration of aortic pseudoaneurysm located at isthmus, left subclavian artery could be closed to avoid endoleakage if necessary. Although the incidence of paraplegia is lower after conventional thoracic artificial vessel implantment, paraplegia might be caused by closure of intercostal artery after the implantation of stent graft. Shorter stent graft is preferred because lesion range of thoracic aortic pseudoaneurysm is small. With the aim of closing laceration, design of the length of graft should avoid obstructing 3 couples or above of intercostal artery as well as radical artery, 85 % of which is arising from the level from T9 to L2, with highest of T5 and lowest of L4; therefore distal should not exceed the vertebral level of T7 so as to bring less effect to blood supply of spinal cord and prevent paraplegia. Stent displacement could block branch vessel access at tumor distal, such as intercostal artery, celiac trunk, renal artery, etc. lead to key viscera ischemia or nerve injury causing non-expected injuries or even failure as well as serious complications like paraplegia, renal failure, intestinal necrosis, etc. Main causes for displacement are: Stent is not adherent due to over-short aneurysm neck, trumpet-shape aneurysm neck and plaque on inner wall of aneurysm neck; badly twisted lesion area, inappropriate size of stent, poor bearing and bending flexibility of the stent, etc. Systolic pressure should be reduced to and maintained at 80- 90 mmHg via controlled hypotension before release of the stent, so as to reduce the impact of blood flow on stent. In order to reduce the risk of distal limbs embolization, it is recommended to release tourniquet in turn at the both sides of femoral artery incision after releasing stent and withdrawing delivery system, and observe closely gush of blood; and eliminate embolization likely existed in the vessel if there is any gush of blood. Occlusion in stent accounts for 17 % of all intravascular stent graft exclusion cases. The causes might be poor dilation, deformation, twisting and full extension failing after stent release; or reduction of collateral vessels after stent graft release; furthermore, endothelial cells are proliferative and adherent to bare stent, which is also seen in the stent interior and perivascular space where the stent was placed. Stent graft with antithrombin and normative postoperative anticoagulant therapy can effectively prevent the occurrence of occlusion [17]. After operation, hemorrhage, infections and formation of skin scar should be attended to, and cover rupture or stent fracture might happen.

## Conclusion

Covered-stent intravascular repair had a good short- and medium-term effect, and can serve as the alternative for some aortic operations. In summary, interventional therapy for pseudoaneurysm is an efficacious therapy features minimal invasion, safety and good prospects.

## Consent

Written informed consent was obtained from the patient for publication of this Case report and any accompanying images. A copy of the written consent is available for review by the Editor-in-Chief of this journal.

## Abbreviations

APA: Aortic pseudo-aneurysm
TEVER: Thoracic Endovascular Aortic Repair
